# Behavioral evaluation in children with obstructive sleep disorders

**DOI:** 10.1016/S1808-8694(15)30044-6

**Published:** 2015-10-19

**Authors:** Sandra Fumi Hamasaki Uema, Marla Vargas Rodrigues Vidal, Reginaldo Fujita, Gustavo Moreira, Shirley Shizue Nagata Pignatari

**Affiliations:** a Post-Graduate student. MSc from the Discipline of Pediatric Otolaryngology - - UNIFESP (MD).; b Specializing Physician from the Pediatric Otolaryngology Department - UNIFESP-EPM, MD, Otolaryngologist.; c Assistant professor from the pediatric otolaryngology department - UNIFESP-EPM, MD, Otolaryngologist.; d MD, Sleep Institute - UNIFESP-EPM.; e Associate professor and head of the pediatric otolaryngology department UNIFESP-EPM, MD, Otolaryngologist. Federal University of São Paulo.

**Keywords:** Behavioral, Children, Sleep, Disorder, Obstructive

## Abstract

**Aim:**

The purpose of this study was to evaluate the behavior in children with obstructive sleep disorder.

**Study design:**

Prospective study.

**Material and method:**

Children's parents (4 to 18 years old) completed the CBCL4/18 (Child Behavior Checklist) in the period of January to July 2005.

**Results:**

In the group, 12 (60%) were males and 8 (40%) females; the total problem score was abnormal in 5 children (25%); introversion was affected in 2 children (10%); extroversion in 5 children (25%). The scales of individual syndromes were abnormal in between 0 and 20% of patients. The individual scales most frequently affected were: total competency (20%), somatic complaints (10%), social problems (10%) and aggressive behavior (10%).

**Discussion:**

This study demonstrates high prevalence (25%) of abnormal behavior. Although widely reported as a common complication of pediatric OSAS, behavioral and neurocognitive disorders have mostly been inferred in several cases and studies. There are few recent studies using standardized assessment to evaluate these alterations. Our study presents preliminary results of the behavior of children with obstructive sleep disorders.

## INTRODUCTION

Obstructive sleep disorders are relatively frequent in the pediatric population and include primary snoring (PS) and the obstructive sleep apnea syndrome (OSAS). PS clinical features respiratory noise, however preserving sleep architecture, alveolar saturation and hemoglobin oxygen saturation. It is frequent in childhood and affects about 7 to 9% of the children between one and ten years of age^1^; some authors estimate that over 12% of children are affected with PS. Notwithstanding, Childhood OSAS features snoring or respiratory noise during sleep, associated to hypoxemia and hypercarbia, sleep disorders or daily symptoms such as oral breathing, aberrant behavior and excessive daily sleepness^2^. Although the literature bears inaccurate incidence figures, present evidence suggests that childhood OSAS affects between 1 and 2% of children^3^. OSAS in children results in clinically significant consequences, including growth delay^4^, right and left ventricular dysfunction^5^, ^6^ and learning and behavioral disorders^7^, ^8^, ^9^, ^10^. Adenoid and palatine tonsil hypertrophy is the most common cause of childhood OSAS, and this diagnosis is best confirmed by polysomnography. There is growing evidence supporting the association between OSAS, attention deficit and hyperactivity disorder (ADHD)^8^. Previous studies have shown an approximate 3 fold increase in behavior and attention disorders in children with obstructive sleep disorder^8^, ^11^, ^12^, ^13^ and recent standardized behavior assessment studies have supported these findings^14^, ^15^. The present study aims at assessing the behavior of children with obstructive sleep disorder.

## MATERIALS AND METHODS

Parents of 4 to 18 year old children were called up to the Oral Breathing Center - UNIFESP-EPM from January to July 2005. The children underwent a clinical interview, complete otorhinolaryngological physical exam (oral exam, anterior rhinoscopy and otoscopy), nasofibroscopy (adenoid hypertrophy) and polysomnography. Children with cranial-facial syndromes, neuromuscular diseases, learning, behavior and psychiatric disorders were excluded from the study. Parents of 20 children answered the CBCL/4-18 questionnaire. We included children with polysomnographyc diagnosis of primary snoring and obstructive sleep apnea and hypopnea syndrome. Filling out the questionnaires The CBCL/4-18 (Child Behavioral Checklist), an inventory of children and teenager behavior in the Brazilian version, is but a behavior inventory created by Achenbach (1991a), that assesses the social competence and behavior disorders in children and teenagers between 4 and 18 years, based on information provided by the parents, thus yielding their social and behavioral profile. It takes from 30 to 60 minutes to answer the questionnaire, depending on the informant's level of understanding. It is a test with 113 specific items on children behavior. Considering the last 6 months behavior, each item is scored as follows: false or totally absent = zero; partially true or sometimes present=1; or frequently present behavior=2. The CBCL/4-18 also bears 35 questions about the child's competence at school, in a social context and activities, which are scored based on the quantity and quality of 6 year or older children's participation (total competence). The social profile is based on three individual scales: activities, sociability and schooling, the sum of these scales yields the social competence. The behavioral profile is made up of 9 individual scales: 8, which are always present and 1 optional. The first 8 behavioral scales correspond to the following syndromes: I. Shyness; II. Somatic Complaints; III. Anxiety/Depression; IV. Problems with social interaction; V. Thinking problems; VI. Attention deficits; VII. Delinquent behavior; and VIII. Aggressive behavio; while the optional scale corresponds to the ninth syndrome: sexual problems. The I, II and III Scales are called Introversion scales when considered together, while scales VII and VIII are called Extroversion Scales when grouped. The sum of the crude scores obtained from the individual behavioral scales corresponds to the Sum of the Behavioral Problems (crude score). The scores are assigned according to Achenbach's manual - 1991a. CBCL/4-18 is scored in order to obtain a total problem score: introversion group scoring (shyness, somatic complaints, anxiety and depression), extroversion group scoring (delinquent or aggressive behavior), and scores of individual syndrome scales (shyness, somatic complaints, anxiety or depression, social problems, thought problems, attention deficits, delinquent and aggressive behavior).

## RESULTS

Parents from 20 children filled out the CBCL, corresponding to 12 boys and 8 girls. Table 1 shows scores for total problem, introversion and extroversion. Total problem was not normal for 5 children (25%), the introversion group was not normal for 2 patients (10%), the extroversion group was not normal for 5 patients (25%) and the individual syndrome scales varied from 0 to 20% in abnormality rate. Table 2 depicts the most affected individual problem scales to be the following: total competence (20%), somatic complaints (10%), social problems (10%) and aggressive behavior (10%).

**Table 1:** Children with CBCL 4/18 grouping alteration (Crude scores).

Groups	N total=20 (100%)
Total problem score	N=5 (25%)
Introversion	N=2 (10%)
Extroversion	N=5 (25%)

**Table 2:** Children with alterations in the CBCL 4/18 individual scales (crude scores)

Individual scales	N total=20 (100%)
Total competence	N=4 (20%)
Somatic complaints	N=2 (10%)
Social problems	N=2 (10%)
Aggressive behavior	N=2(10%)

## DISCUSSION

This study shows high abnormal behavior prevalence (25%). Although it has been largely mentioned as a common complication of childhood OSAS, behavior and neurocognitive disorders have been inferred in series of cases in which parents’ reports were the assessment criteria. There are few studies using standardized measures to assess behavior and development disorders. Some papers report that adenotonsillectomy results in significant improvement of both behavior and sleep respiratory disorders^13^, ^16^. Ali et al.^14^ used nocturnal pulse oximetry and video-recording in 66 children with suspicion of sleep apnea, and 66 controls. Three Conners’ Behavior Rating Scale (CRTS) were given to the parents and teachers. Only 7 children (all from the high-risk group) had detectable respiratory disorder, however, high-risk children had significantly higher hyperactivity and attention deficit scores in the Conners’ subscales filled out by their parents. In a study follow up, Ali et al.^17^ repeated the continuous performance test evaluations – a measure of sustained attention in children - in 33 children with suspected sleep apnea. Of these, 12 had respiratory sleep disorder (OSD) in the video recording and pulse oximetry, while 21 children had normal sleep. Age and gender paired eleven of these children with those from the OSD group. 10 other children without past history of snoring formed the control group. After adenotonsillectomy, the OSD group showed mild, but still significant improvement in all CTRS scales, while the snoring group improved only in the hyperactivity subscale. There were no significant changes in any of the CTRS groups. After adenotonsillectomy, there was an improvement in behavior pattern and in continuous performance in both the OSD and snoring groups.

In a study involving 36 children, Goldstein et al. showed a prevalence of 28% of emotional and behavioral problems^18^, and this is in agreement with the findings of the present study. The limitations of this study include small samples and a lack of a healthy children control group. Our study is a preliminary result of the behavioral profile of children with obstructive sleep disorder. CBCL is a valid and comprehensive assessment of children's emotional and behavioral problems intensely used by pediatric psychiatrists, psychologists, pediatricians, special educators, and for the appreciation of problems brought about by child abuse. Behavioral, emotional and neurocognitive difficulties are present in children with OSAS. Larger samples and control groups are necessary to accurately define the behavior of children with obstructive sleep apnea.

## References

[1] Carroll JL, Loughlin GM (1992). Diagnostic criteria for obstructive sleep syndrome in children. Pediatr Pulmonol.

[2] Diagnostic Classification Steering Committe 1990

[3] Brouillette R, Hanson D, David R (1984). A diagnostic approach to suspected obstructive sleep apnea in children. J Pediatr.

[4] Bar A, Tarasiuk A, Segev Y, Philip M, Tal A (1999). The effect of adenotonsillectomy on serum insulin-like growth factor-I and growth in children with obstructive sleep apnea syndrome. J Pediatr.

[5] Tal A, Leiberman A, Margulis G, Sofer S (1988). Ventricular dysfunction in children obstructive sleep apnea: radionucleide assessment. Pediatr Pulmonol.

[6] Amin RS, Kimball TR, Bean JA (2002). Left ventricular hypertrophy and abnormal ventricular geometry in children and adolescents with obstructive sleep apnea. Am J Respir Crit Care Med.

[7] Guilleminault C, Korobkin R, Winkle R (1981). A review of 50 children with sleep apnea syndrome. Lung.

[8] Chervin RD, Dillon JE, Bassetti C, Ganoczy DA, Pituch KJ (1997). Symptons of sleep disorders, inattention, and hyperactivity in children. Sleep.

[9] Gozal D (1998). Sleep-disordered breathing and school performance in children. Pediatrics.

[10] Gozal D, Pope DW (2001). Snoring during during early childhood and academic performance at ages thirteen to fourteen years. Pediatrics.

[11] Owens J, Opipari L, Nobile C, Spirito A (1998). Sleep and daytime behavior in children with obstructive sleep apnea and behavior disorders. Pediatrics.

[12] Rosen CL (1999). Clinical features of obstructive sleep apnea hypoventilation syndrome in otherwise healthy children. Pediatr Pulmonol.

[13] Bulden S, Lushington K, Kennedy D, Martin J, Dawson D (2000). Behavior and neurocognitive performance in children aged 5-10 years who snored compared to controls. J Clin Exp Neuropsychol.

[14] Ali NJ, Pitson DJ, Stradling JR (1993). Snoring, sleep disturbance, and behavior in 4-5 year olds. Arch Dis Child.

[15] Rhodes SK, Shimoda KC, Waid LR (1995). Neurocognitive deficitsin morbidly obese children with obstructive sleep apnea. J Pediatr.

[16] Stradling JR, Thomas G, Warely ARH, Williams P, Freeland A (1990). Effect of adenotonsillectomy on nocturnal hypoxemia, sleep disturbance, and symptoms in snoring children. Lancet.

[17] Ali NJ, Pitson DJ, Stradling JR (1996). Sleep disordered breathing: effects of adenotonsillectomy on behavior and psychological functioning. Eur J Pediatr.

[18] Goldstein NA, Post C, Rosenfeld RM, Campbell TF (2000). Impact of tonsillectomy and adenoidectomy on child behavior. Arch Otolaryngol Head and Neck Surg.

